# Microbiota in the apical root canal system of tooth with apical periodontitis

**DOI:** 10.1186/s12864-019-5474-y

**Published:** 2019-04-04

**Authors:** Wenhao Qian, Ting Ma, Mao Ye, Zhiyao Li, Yuanhua Liu, Pei Hao

**Affiliations:** 1 Shanghai Xuhui District Dental Center, Shanghai, 200032 China; 2 Key Laboratory of Molecular Virology and Immunology, Institut Pasteur of Shanghai, Chinese Academy of Sciences, Shanghai, 200031 China

**Keywords:** Apical periodontitis, 16S rRNA sequencing, Root canal treatment, Bacterial community, Healthy control

## Abstract

**Background:**

Apical periodontitis (AP) is essentially an inflammatory disease of microbial etiology primarily caused by infection of the pulp and root canal system. Variation of the bacterial communities caused by AP, as well as their changes responding to dental therapy, are of utmost importance to understand the pathogensis of the apical periodontitis and establishing effective antimicrobial therapeutic strategies. This study aims to uncover the composition and diversity of microbiota associated to the root apex to identify the relevant bacteria highly involved in AP, with the consideration of root apex samples from the infected teeth (with/without root canal treatment), healthy teeth as well as the healthy oral.

**Methods:**

Four groups of specimens are considered, the apical part of root from diseased teeth with and without root canal treatment, and wisdom teeth extracted to avoid being impacted (tooth healthy control), as well as an additional healthy oral control from biofilm of the buccal mucosa. DNA was extracted from these specimens and the microbiome was examined through focusing on the V3-V4 hypervariable region of the 16S rRNA gene using sequencing on Illumina MiSeq platform. Composition and diversity of the bacterial community were tested for individual samples, and between-group comparisons were done through differential analysis to identify the significant changes.

**Results:**

We observed reduced community richness and diversity in microbiota samples from diseased teeth compared to healthy controls. Through differential analysis between AP teeth and healthy teeth, we identified 49 OTUs significantly down-regulated as well as 40 up-regulated OTUs for AP.

**Conclusion:**

This study provides a global view of the microbial community of the AP associated cohorts, and revealed that AP involved not only bacteria accumulated with a high abundance, but also those significantly reduced ones due to microbial infection.

**Electronic supplementary material:**

The online version of this article (10.1186/s12864-019-5474-y) contains supplementary material, which is available to authorized users.

## Background

Apical periodontitis (AP) is an endodontic disease, with inflammatory lesion around the apex of a tooth root which is usually caused by microbial invasion of the root canal system [1, 2]. It is mostly the outcome of untreated dental caries when the root canal system is infected by oral microbiota [3]. This root canal infection can become symptomatic and evolve to severe spreading and sometimes life-threatening abscesses [3]. Treatment of AP involves antibiotic therapy and root canal treatment (RCT, also called endodontic therapy) or extraction of the diseased tooth to eliminate the source of infection. Conventional endodontic therapy was reported yielding a highly successful outcome, with the contribution of enhanced diagnostic tool, antibiotics, and advances in biomaterials [4]. Endodontic instrumentation alone can’t achieve a sterile condition due to the anatomical complexities of root canals and limitations of medicaments. Initiation of empirical therapy with antibiotics is highly recommended for lesion sterilization and tissue repair. As examples, from the early used EDTA [5], to sodium hypochlorite [6], chlorhexidine and octenidine hydrochloride [7], as well as the triple antibiotic paste (combination of metronidazole, ciprofloxacin, and minocycline) [8, 9], are widely applied and tested effective for removing microbiome. Even so, quite alarming numbers in the persistence of AP were reported in literature [4], since complete elimination of microorganisms is not always obtained. The spectrum of the antimicrobial agent, based on experience or even microbial susceptibility testing [3], mostly can only cover partial of the pathogenic community (a set of species, usually organized in multispecies biofilm) [10, 11]. This highlights the urgency to uncover the composition of the microbial community involved in AP and hence to elucidate the “unit of pathogenicity” of AP comparing to normal conditions.

Early studies of the microbiota in the root canals of teeth with AP were conducted through broad-range culture/biochemical methods, by which only cultivable and predominant bacteria were reachable, with the risk of missing keystone species [12, 13]. The adoption of 16S rRNA-based microbial diversity analyses allows the detection of a large scale bacterial communities in endodontic infections, including the cultivable/uncultivable and even the uncharacterized bacterial community members, as well as the low abundance taxa [14]. Growing evidence showed that not only one but several species contribute to endodontic infection, which interact one with the other to give rise to disease.

Bacteria located in the apical part of the infected root canals are arguably directly involved in the AP pathogenesis. Researchers were challenged in sampling procedures when evaluating the endodontic microbiota was performed. Paper points are mostly used in early studies, but it is widely accepted that limited region of the root canal can be involved. Gryogenic grinding technique was widely used recently, where the root apex fragment was sectioned and together with the surrounding tissue was cryogenically ground to collect the microbiota samples [15].

Based on the 16S rRNA technique, complex bacterial populations were reported present in AP individuals, mostly classified within phyla of *Proteobacteria*, *Firmicutes*, *Bacteriodites*, *Fusobacteia*, and *Actinobacteria* [12, 16–23], where *Proteobacteria*, *Firmicutes* and *Bacteriodites* were always found prevalent. With 10 patients (10 extracted teeth) involved, Siqueira’s early study identified 187 phylotyes belonging to 84 genera and 10 phyla in apical root canal infections, where *Proteobacteria* and *Firmicutes* together occupied about two thirds of all sequences obtained [16]. Later, in Vengerfeldt’s work [18], *Firmicutes* and *Bacteroidetes* were observed prevalent in chromic apical periodontitis. Their work has revealed many of the known root canal pathogens including gram-negtive anaerobes such as *Prevotellasp*, *Porphyromonassp*, *Fusobacteriumsp*, *Tannerellasp*, and *Pyramidobacter piscolens* as well as gram-positive *Dialistersp*, oral spirochete *Treponema socranskii*, *Solobacterium moorei*. They also noted an increase of *E.faecalis* after treatment failure. Most recently, Rocas [24] found *P.endodontalis*, *Dialisterinvisus, Olsenellauli* and *F.nucleatum* were most frequently detected in their asymptomatic AP cases.

We note that the profile of the microbial community around the apical part of the root in AP differed at different taxonomic levels with respect to labs (mainly due to geographic differences) [25, 26], and individual diversity was commonly observed even in a single study. Furthermore, researchers aimed to uncover the microbial composition and diversity, and focused on the taxons (OTUs, species or genus) which are predominant in AP to understand the pathogenesis of AP and establish effective antimicrobial therapeutic strategies [15]. However, no microbial profile of the root apex was available for normal condition. This might be due to the fact that it is difficult or even impossible to collect the extracted tooth for normal condition. Thus, questions could arise: is there microbiome located around the root apex in the normal condition? Without the answer of this question, it is ambiguous to consider the microorganisms predominantly present in AP as putative pathogens. In fact, scientists in this field did be challenged for clarifying whether more-virulent communities develop right from the beginning of the infection process or are a result of a shift in the community composition due to environmental changes [3]. Contribution from the side of the normal condition (or close to the normal condition) will aid in unearthing this pathogenic process of AP. Most recently, Coleman and colleagues [21] considered the supragingival samples (supragingival buccal biofilm scraping) from healthy teeth as reference in their work, and revealed an increased abundance of Gram-negative (*Prevotella*, *Fusobacterium*, *Treponema*, *Veillonellaceae*, *TG5* (*Synergistetes*) bacteria and a decreased abundance of Gram-positive (*Actinomyces*, *Corynebacterium*) bacteria in the diseased tissue samples. Bacteria decreased in diseased cases were emphasized in this study, somehow indicating the dysbiosis caused by AP. However, the bacterial community located in the supragingival buccal biofilm is too limited to represent the root canal system since distinguished variation of microbial composition and diversity were observed in different regions of human oral cavity [25, 26].

We therefore plan our research in two ways: i) collecting 16S rRNA samples from both healthy wisdom teeth (extracted in case of becoming impacted) and the oral sample (through rubbing a sterile cotton swab across the buccal mucosa) for each contributor, by which to investigate the microbial community close to normal condition and uncover the relationship between oral and the root apex microbiota; ii) collecting root apex specimen from AP to identify the key bacteria relevant to the etiology and pathogenicity of AP. We also involved the diseased teeth failed after conventional root canal therapy (RCT) to explore the effect of RCT on microorganisms in the tooth root canal system.

## Materials and methods

### Subjects population

We considered the teeth and oral samples from AP patients (31) and healthy controls (9). From the 31 patients, we collected 8 teeth failed after RCT (labeled as RCT) and 23 teeth without RCT (labeled as NRCT), where each patient contributed one. 10 healthy teeth (labeled as HT) were obtained through common surgical extractions of supernumerary or wisdom teeth, where one healthy control contributed two and others each contributed one. We also collected 9 oral samples from healthy people who contribute the wisdom teeth (labeled as HO). The diseased teeth were presented with a 2–8 mm AP lesion as determined radiographically, and clinically classified as LSA (maximum diameter > =5 mm) and SSA (maximum diameter < 5 mm) according to the area of AP lesion. The inclusion criteria for RCT failure cases: i) previous root canal treatment or retreatment performed at least more than 2 year before; ii)the lesion remained the same size or increased as compared with radiographs taken immediately after the initial treatment/retreatment. Exclusion criteria of AP teeth: i) direct connection of the apical lesion to the oral cavity due to lack of gingival tissue, vertical root fracture and fistula; ii) previous surgery on the apical root; iii) treated with antibiotics within one month.

### Sampling procedure and clinical data collection

Oral specimen was collected from individual by rubbing a sterile cotton swab across the buccal mucosa and frozen for storage at − 20 °C. Diseased teeth with/without RCT were extracted because of being unrestorable, while wisdom teeth (healthy controls) were extracted in case of being impacted. The apical part of the tooth root was resected at 2 mm from the root end using a sterile Zekrya FG 28 mm bur, as reported in [19]. Before the tooth extraction surgery, the oral cavity was rinsed with 0.12% chlorhexidine for 2 min and the area to be operated was gently scrubbed with the same solution. Care was taken to avoid saliva contamination of the surgical site during the whole surgery. The root apex specimen was soon placed in a sterile flask and immediately frozen at − 20 °C. The root apexes were finally crushed in a 6750 freezer mill (Spex, Metuchen, NJ, USA), at the liquid nitrogen temperature, as reported in [15]. Apical root powder samples were stored frozen at − 20 °C.

### DNA extraction and 16S rDNA amplicon pyrosequencing

Apical root powder samples and oral samples were treated with the Fast DNA SPIN extraction kits (MP Biomedicals, Santa Ana, CA, USA), following the manufacturer’s instructions, to extract the total DNA, and stored at − 20 °C prior to further analysis. The extracted DNAs were then quantified and qualified using a NanoDrop ND-2000 spectrophotometer (Thermo Fisher Scientific, Waltham, MA, USA) and agarose gel electrophoresis, respectively. PCR amplifications were performed using bacterial domain-specific primers, targeting variable regions V3-V4 of the 16S rRNA gene, using primers 338F (5’-ACTCCTACGGGAGGCAGCA-3′) and 802R (5’-GGACTACHVGGGTWTCTAAT-3′). Details of the PCR components and the thermal cycling procedures were described in [27]. Purification and quantification of the PCR amplicons were done by Agencourt AMPure Beads (Beckman Coulter, Indianapolis, IN) and PicoGreen dsDNA Assay Kit (Invitrogen, Carlsbad, CA, USA), respectively. After that, equal amounts of the amplicons were pooled, and 2 × 250 bp paire-end sequencing was performed using the illumina MiSeq platform with MiSeq Reagent Kit v3.

### Sequence analysis

Sequences were processed with QIIME following an established illumima sequencing analysis pipeline [28]. Briefly, paired-end reads were firstly joined using SeqPrep method (https://github.com/jstjohn/SeqPrep) where minimum overlap between paired reads is set as 5 bps. Demultiplexing was then performed to assign the assembled reads to individual samples according to the barcodes and filtered through the default strategy for quality filtering of illumina data described in [29]. An open-reference clustering [30] was then used to approach OUT picking, where the Greengenes database (the Greengenes 13_8 97% OTU representative sequences) and denovo clustering of unmapped sequences were combined to build the reference database. Sequences were queried against the reference database and assigned to the reference OTUs if the identity is not less than 97%. The remainder of the sequences that fail to hit the reference database were then be clustered using UCLUST [31] for denovo reference OTUs, and assigned to the new reference OTUs. An OTU table was further generated to record the abundance of each OTU in each sample and the taxonomy of these OTUs. This has singleton OTUs (or OTUs with a total count of 1) removed, as well as OTUs whose representative (i.e., centroid) sequence couldn’t be aligned with PyNAST [32].

The OTU table was then used to estimate the community richness (chao1 and OTUs) and diversity indices (Shannon index and simpson index) for individual sample, using R packages. Shannon index and simpson index were calculated using the function *diversity* in package *vegan*; Chao1 was obtained through chao1 in package *fossil.*

### Statistical analysis

All statistical analysis starts from the raw counts of the OTU table, and taxa of the same type were summarized at the phylum, class, order, family and genus levels. For comparisons between samples/groups, abundance of each OTUs were normalized by a down-sampling way, where the number of sequences of individual sample was reduced to the minimum of all the 50 samples by random sampling. To identify the essential OTUs relevant to AP, the OTUs were firstly filtered to remove the OTUs present in less than 10% of all samples. Wilcoxon test [33] was then used to perform differential analysis between different groups (e.g. HO, HT, NRCT and RCT). *P*-values were adjusted by Storey’s Q value [34]. Significant OTUs were selected using q-value 0.05, 2 fold-change as well as the Q3 quantile from the two groups involved in any comparisons. The Q3 quantile threshold was employed to filter the candidates if the 75th quantile of the high-level group is less than 10 in NRCT versus HT at OTU level and less than 100 at Genus level.

## Results

### Coverage, richness and diversity

**Fig. 1:**
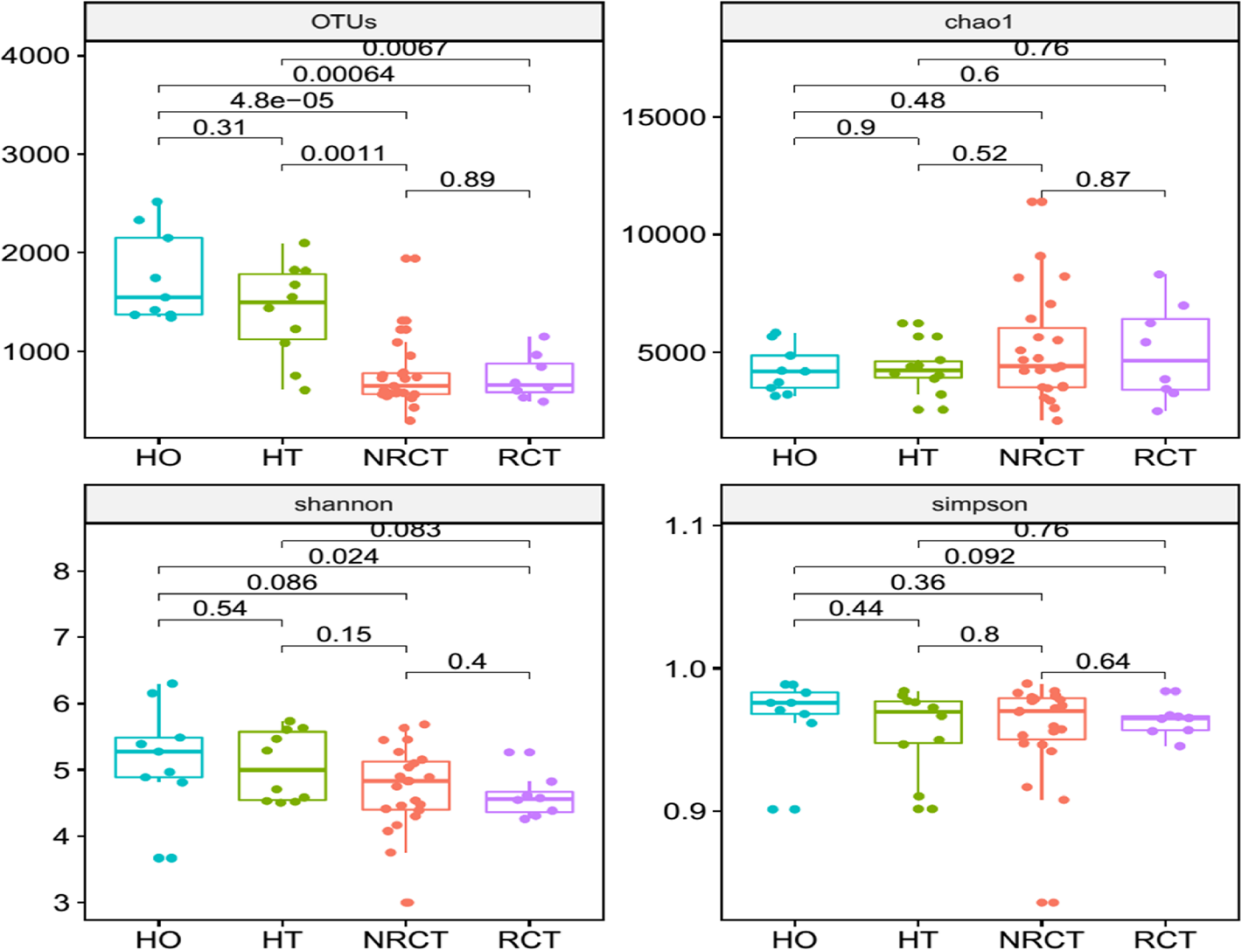
Bacterial community richness (observed OTUs and chao1) and diversity (Shannon and Simpson index) in the groups of AP (NRCT), AP failed after RCT treatment (RCT), healthy control of root apex (HT), as well as the oral healthy control (HO)

We have 50 16S samples in total including diseased and healthy teeth samples as well as oral samples. The number of the valid reads from 16S RNA sequencing for OTUs detection is ranging from 29,604 to 68,088. 43,839 OTUs in total appear across 50 samples, while the number of OTUs in single sample ranges from 918 to 4436. To study the changes of the richness and evenness of individual samples in terms of locations and healthy status, we calculated the richness (OTUs and Chao1) and diversity indices (Shannon and Simpson), and compare them from different groups (HO, HT, NRCT and RCT). Comparing to the diseased samples, higher richness and diversity were observed in healthy samples, including healthy oral and healthy teeth (Fig. 1). In particular, the numbers of the OTUs in healthy samples (HT and HO) were significantly higher than the diseased samples (RCT and NRCT). This indicates that the balance of the microbial community of the root apex was changed due to the disease as well as RCT treatment. Dynamically saying, i) healthy tooth host plentiful of bacteria (both richness and diversity), ii) AP significantly reduced the OTU richness (*P* values are 0.0011 and 0.0067 for NRCT versus HT and RCT versus HT, respectively) and made the community purer; iii) and after that, RCT treatment further slightly increased the OTU number but reduced the diversity. We therefore speculate that microbial infection in AP caused part of the bacteria increased sharply while part of some other bacteria decreased or even disappear. In other word, there might be some bacteria becoming predominant due to infection. For this, we sorted the OTUs based on their abundance, and calculated the fraction of the abundance accumulation of the top OTUs in the total abundance of the whole sample. Dynamics of the fraction with the increasing of the number of the top OTUs showed that the fraction in NRCT and RCT groups dramatically increased and close to a steady state much earlier than healthy groups (Additional file 1: Figure S1). This indicates that much fewer bacteria in NRCT and RCT have very high abundance and dominate the bacterial community (the fractions close to 85, 83, 75 and 72% at top 100 OTUs in RCT, NRCT, HT and HO, respectively). Root canal treatment has pulled the richness (OTUs) back slightly (NRCT compared with RCT, from 764 ± 352 to 740 ± 229 in Fig. 1), but it is still far away from that in the healthy status (from 764 ± 352 to 1410 ± 484 in Fig. 1). Furthermore, the lower diversity of RCT as well as the top 100 OTUs occupied 85% of the whole abundance (Additional file 1: Figure S1) somehow told that root canal treatment was not able to change the dominant phenomena.

### Global view of the bacterial communities

**Fig. 2:**
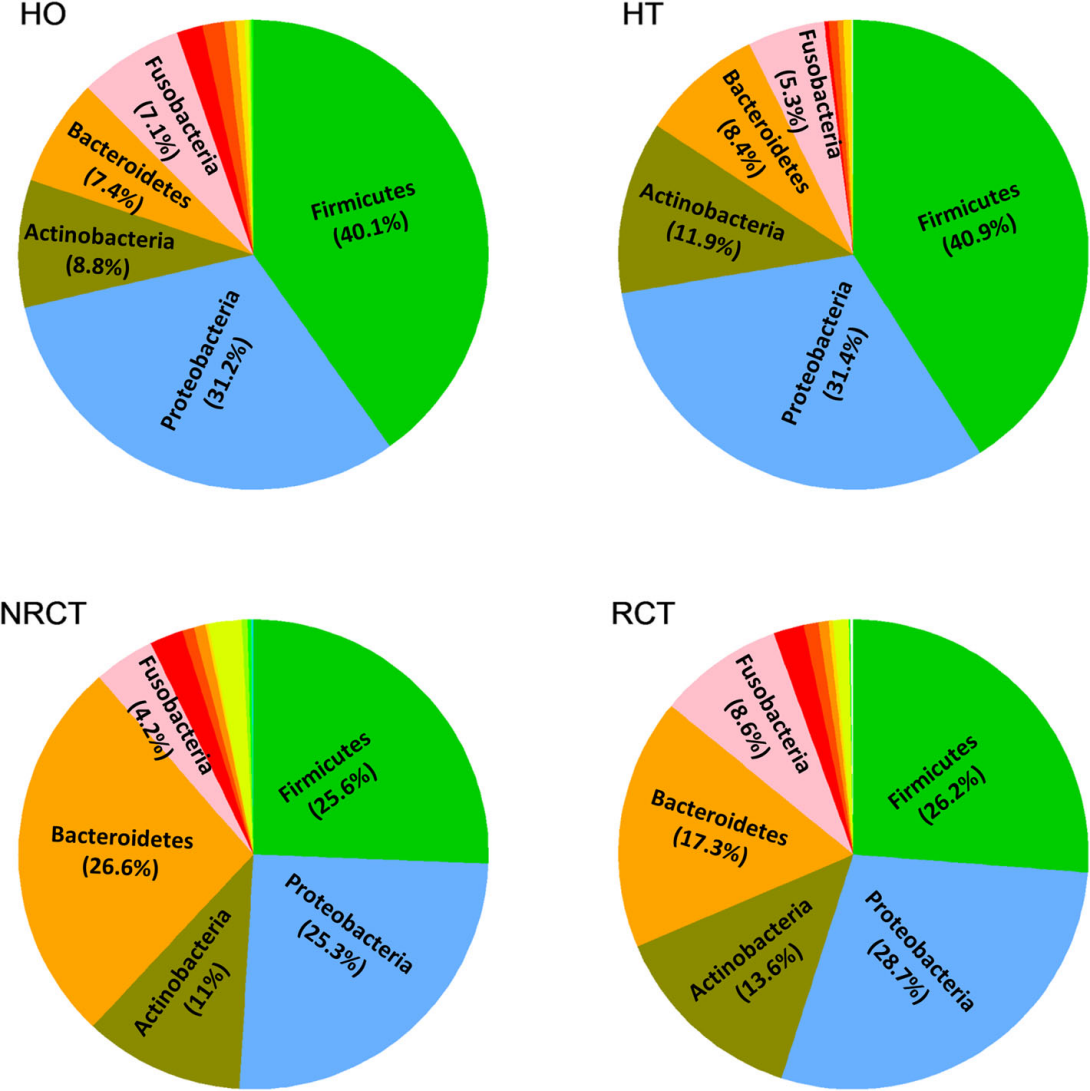
Piechart of the distributions of the species in individual samples at phylum level. NRCT, AP without RCT treatment; RCT, AP failed after RCT treatment; HT, Healthy control of root apex; HO, the oral healthy control

26 phyla present in the 50 microbiota samples. From a global view, the most abundant phyla are *Firmicutes*, *Proteroidetes* and *Bacterioidetes*, followed by *Actinobacteria*, *Fusobacteria*, *Spirochaetes*, *Synergistetes*, *Acidobacteria*, *TM7*, *Cyanobacteria*, *Tenericutes*, *Chloroflexi*, *SR1*, *Elusimicrobia*, *GN02*, *Planctomycetes*, *Gemmatimonadetes*, *[Thermi]* and *Nitrospirae*. Percentages differed due to individuals as well as location, healthy status or treatment (Fig. 2, details see Additional file 1: Figure S2). Basically, bacterial distributions at phylum level did not show big difference between two healthy groups (HO and HT). *Firmicutes* is the most abundant in healthy samples, averaged at round 40% in both teeth and oral, while it dropped to around 26% in diseased samples (NRCT and RCT). On the contrary, *bacterioidetes* jumps to the top 1 in NRCT (26.6% in NRCT from 11.9% in HT) and a slight decrease in RCT. Although root canal treatment failed in RCT, it has changed the percentage of *Bacterioidetes* (from 26.6 to 17.3%), *Fusobacteria* (4.2 to 8.6%) and *Actinobacteria* (11 to 13.6%).

Clustering results using tSNE (implemented by an R package Rsne [35]) at OTU level showed that oral samples (HO) samples are basically separated from teeth samples(HT, NRCT and RCT), and healthy teeth (HT) can also be distinguished from teeth without root canal treatment (NRCT), see Additional file 1: Figure S3. However, teeth samples failed after RCT treatments are mixed with other samples.

### Bacterial communities around healthy root apex

**Fig. 3:**
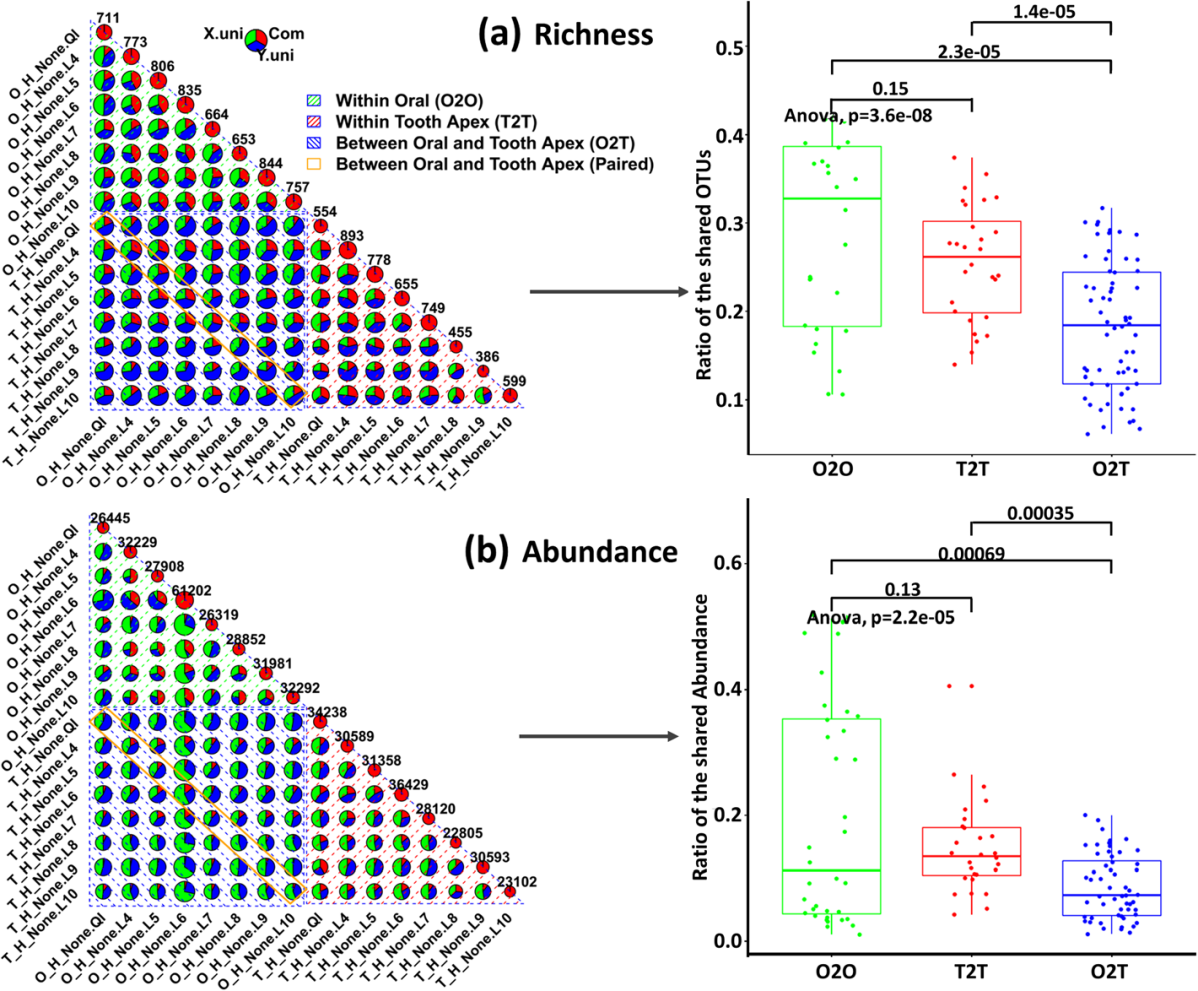
Overlaps of the richness (**a**) and abundance (**b**) between paired root apex and oral samples from the same patient. Upper panel: piecharts to show the unique and common OTUs (richness) or abundance between two samples (only the patients who contributed both oral and tooth samples were considered). X.uni represents the OTU or abundance unique in the sample from X axis; Y.uni represents the OTU or abundance unique in the sample from Y axis; Com means the OTU or abundance shared in two samples. Bottom panel: boxplots to show the shared OTUs and abundance with respect to location (oral and tooth). O2O represents shared OTUs or abundance within oral samples; T2T represents shared OTUs or abundance within tooth samples; O2T represents shared OTUs or abundance between oral and tooth samples

As stated above, we did identify quite a number of bacterial community round the healthy tooth apex (1410 ± 484 OTUs, 120 ± 49 Genera). This gives some clue that the root canal system itself might not initially clean. On the contrary, there are microorganisms commensal around the tooth apex. That means the bacteria appear in the root apex will not work as a pathogen even if they are predominant. Meanwhile, we observed a very close distribution of taxa at phylum level to the healthy oral case (Fig. 2). We next go deep into the OTU level to investigate the relationship of the microbiota between root apex and oral. 16 paired 16S samples from 8 healthy individuals, each contributed one wisdom teeth (HT) and one oral sample (HO), were used for comparisons in two ways: i) differential analysis based on the OTUs between HT and HO, and ii) similarity based on abundance and richness within and between HT and HO. Paired Wilcox test combined with Stoery’s FDR adjustment was used for differential analysis. However, no significant OTUs are identified. For the later way, we calculated the overlaps and uniqueness for every pair of samples from between or within HT and HO. For any two samples from HT and HO, we counted the shared OTUs and those presented uniquely in each samples, and showed it in one piechart in Fig. 3. We then collected the calculated the ratio of the shared OTUs in the piechart for each two samples, which were used to estimate the difference between-group-samples (O2T, overlaps between oral and tooth samples) and within-group-samples similarity (O2O, overlaps between samples from oral; T2T, overlaps between samples from teeth). We did the same way on abundance, but focused on the shared abundance between two samples. Significant difference of the pair-wise overlaps was observed between within oral (O2O), within tooth apex (T2T) and between oral and tooth apex (O2T). Similarity within oral samples is higher than that of within tooth apex samples, and within-group (O2O, T2T) similarity is higher than between-group (O2T) samples. In particular, paired oral and tooth apex samples (oral and tooth samples from the same individual) did not show higher ratio of overlaps compared to non-paired samples. In the abundance case (Fig. 3b), much lower ratio of overlaps was found, and the pair-wise similarity within tooth apex is averagely higher than within oral samples.

### Changes of bacterial communities due to apical periodontitis

From above, we found the changes of richness, diversity as well as distribution due to geographical location (oral and teeth), healthy state (diseased versus healthy) and therapy (RCT and NRCT). Next, differential analysis at OTU level and genus level was done for NRCT versus HT, HT versus HO, and RCT versus NRCT, respectively.

**Fig. 4:**
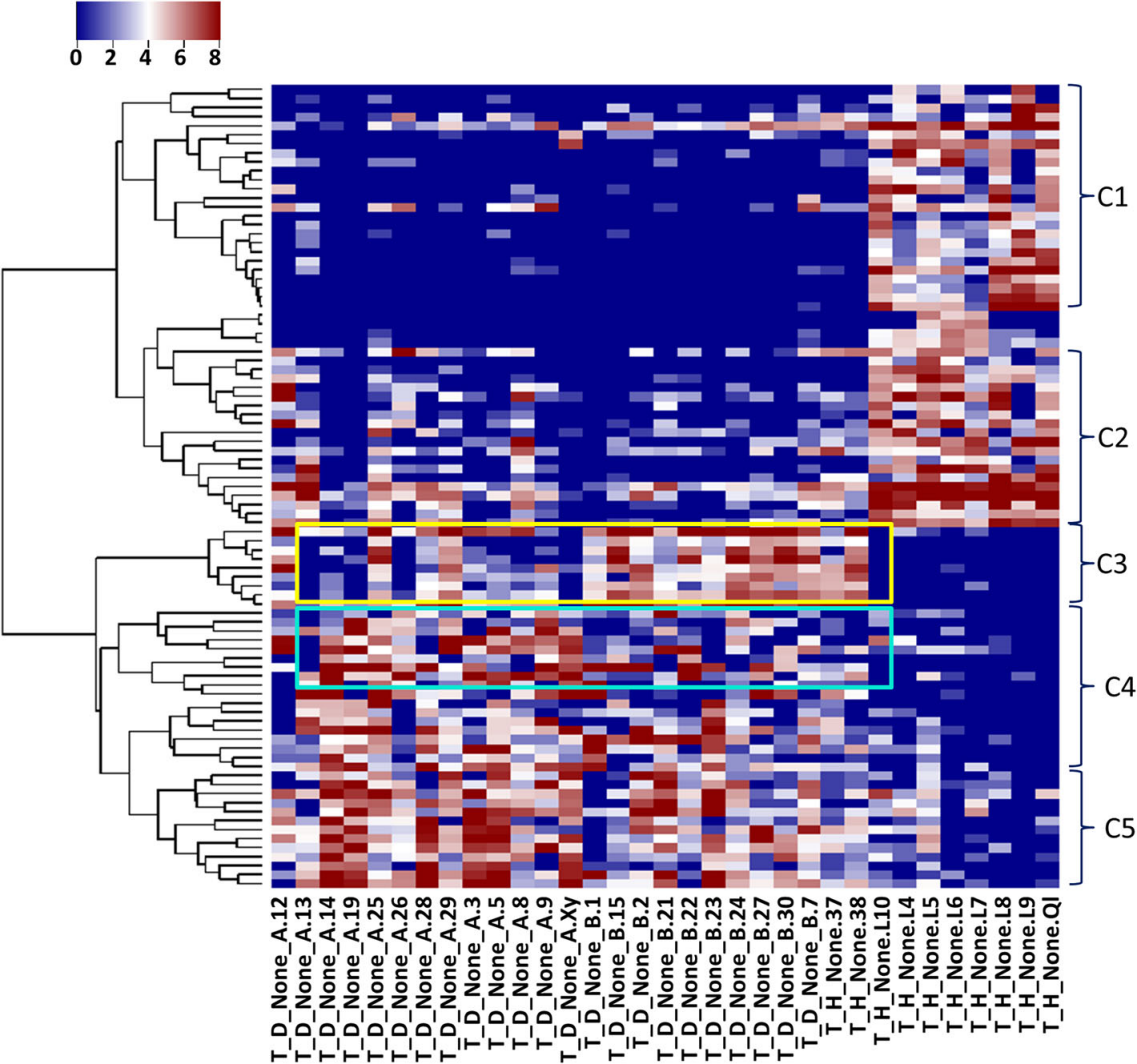
Heatmap of the key OTUs identified through comparison between AP (NRCT) and health control (HT). Key OTUs are clustered into 5 groups, C1~C5 which differed at their variation patterns due to healthy and disease as well as the subtypes (A and B) in the AP samples, detailed OTUs see Additional file 3

**Fig. 5:**
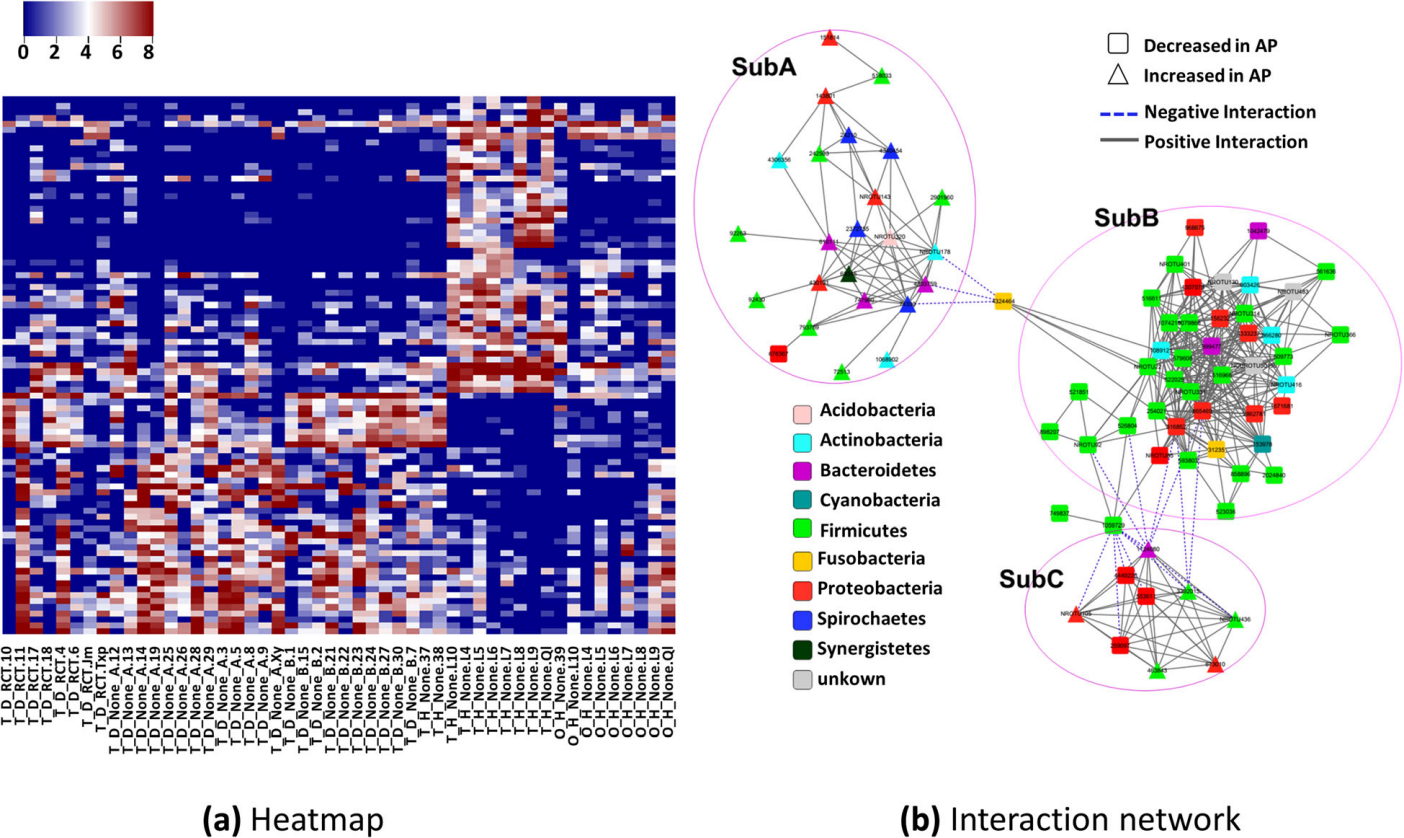
Heatmap and their interactions of the key OTUs identified through comparison between AP (NRCT) and health control (HT). **a** heatmap of the key OTUs across all 50 samples. **b** Interaction network of the key OTUs is constituted with two parts: SubA, where increased OTUs are inter-connected; SubB, where decreased OTUs are inter-connected; SubC: increased and decreased OTUs interacted

We identified 89 significant OTUs in total, details see Additional file 2. 49 of them were significantly decreased in the AP samples compared to healthy teeth. These OTUs are basically from genera of *Streptococcus*, *Haemophilus* (*parainfuenzae* in particular), *Actinomyces*, *Granulicatella, Leptotrichia*. The species including *subflava*, *dispar*, *nanceiensis*, *melaninogenica, mucilaginosa were found*. Almost all of these OTUs have a high abundance in the healthy teeth, while very low abundance was observed in AP teeth (close to zero). These OTUs were clustered into two groups using *hclust* in R, C1 and C2 in Fig. 4. C1 showed very high abundance in healthy, but almost empty in NRCT, while OTUs in C2 were found with a low level, low level of these OTUs were also observed in the oral control and RCT group (Fig. 5a). This indicates that there exists high abundance of bacterial community specific in healthy teeth, but killed due to AP, and they are not recovered in most of the RCT samples. In literature, researchers focused on the abundant bacteria appeared in the AP teeth to identify the pathogenic bacteria for AP. No report was found to describe the bacterial profile around the root apex of healthy teeth, due to limitation of sampling. With the healthy controls in our case, these decreased OTUs told that there did exist plentiful of bacteria and they disappear in the AP cases. We therefore speculate that these abundant bacteria specific in health teeth root might be essential for keeping a healthy environment. Interestingly, *Streptococcus* and *Granulicatella* were considered as putative pathogens due to the fact that they were detected in acute apical abscesses (belong to AP) [3]. They did be detected in our NRCT samples. But far more abundant of them (*Streptococcus* in particular) was found in our healthy teeth. From our experiment, we say that *Streptococcus* and *Granulicatella* are relevant for AP because of their reduction, other than their presence. *Prevotella* was found in both increased and decreased OTUs we identified, while it was widely reported to be increased in literature. Two species in genera of *Prevotella*, *Prevotella naneiensis* and *Prevotella melaninogenica*, are decreased in NRCT compared to HT.

Forty OTUs are significantly increased in AP. These OTUs were grouped into three clusters, i.e. C3, C4 and C5 in Fig. 4. OTUs in C3 showed higher abundance in A than B (A and B are subtypes in NRCT by samples clustering and match with the large lesion and small lesion, respectively with a high accuracy 0.89). The reverse for OTUs in C4. OTUs in C5 were high in both A and B. All of these 40 OTUs showed very low abundance (close to zero) in healthy teeth but very high level in NRCT, including the OTUs in the genera of *TG5*(*Synergistetes*), *Treponema* (*socranskii*), *Sphaerochaeta*, *Burkholderia*, *Schwartzia*, *Dialister*, *Pseudoramibacter*_*Eubacterium*, [*Prevotella*], Slackia, *Bifidobacterium* (*longum*) and [*Mogibacteriaceae*] (family). Among them, *treponema*, *prevotella*, *TG5* are wildly reported to be present in periodontitis [21, 36]. In particular, *Prevotella*, *Treponema*, and TG5 (*Synergistetes*) were observed increased in AP root apex compared to supragingival buccal biofilm (HO samples in our case) from healthy teeth [21]. *Sphaerochaeta* was found in subgingival microbiota with a high abundance from chronic periodontitis patients [37] but in our case it is very low in NRCT and empty in healthy teeth. Very high abundance of *Burkholderia* was found in our NRCT while it is not reported in previous researches. Low abundant *Bifidobacterium* (*longum*) was detected in our NRCT, as described in subgingival in [36, 38], but empty in any of the healthy teeth. We found 2 OTUs from *Pseudoramibacter*_*Eubacterium* genus, both are absent in healthy teeth, while one is with very high abundance and the other is at a low level in NRCT. *Dialister* is frequently recovered from endodontal infections and deep periodontal pockets [3]. *Prevotella* and *Treponema* were also reviewed as factors involving in AP infections [3].

Differential analysis at genus level identified fewer significantly changed genera than that found through OTU level, see Additional file 3 for details, including the decreased *ones Haemophilus, Actinobacillus, Granulicatella, Staphylococcus and Streptococcus, and the increased ones Schwartzia, Slackia, Treponema, TG5, Pseudoramibacter_Eubacterium*. All of them are covered in the genera identified at the OTU level.

### Relationships between the key OTUs

As described above, significant bacteria (OTUs) are identified through comparison between NRCT and HT. We further investigate the relationships between the key OTUs based on the abundance of each OTU across all the samples. Correlations of these OTUs were calculated using R package *corr.test* with the method of Pearson. The OTUs highly correlated (correlations bigger than 0.6 and adjusted *p*-value less than 0.05) were selected to construct a network using cytoscape (Fig. 5b). In the network, OTUs (nodes in Fig. 5b) are partitioned into three sub-networks, basically consisted of increased OTUs (**SubA**), decreased OTUs (**SubB**) as well as both increased and decreased OTUs (**SubC**), respectively. OUT 43244264 (yellow filled box) works as a connection between SubA and SubB. K-core calculations (the strength to be a core of any cluster) of the whole network showed a bimodal distribution wherein most of the OTUs have a very high value of k-core values and others with very low values. Decreased OTUs from the phyla of *Firmicutes*, *Proteobacteria* as well as some unknown species are tightly connected in **SubB**. We say that these decreased species co-varied with respect to location, AP or RCT therapy with equivalent contribution, and built a robust network. This somehow indicates the difficulty to destroy the balance of the bacterial community through the decreased species. In other word, it is hard to recover once the network is broken. This somehow explained the failure of RCT in our case, where decreased OTUs showed no big changes in RCT compared to NRCT (Fig. 5a). Increased OTUs are interacted in **SubA**, but showed weak relationships (fewer connections and smaller correlation values). **SubB** and **SubC** are negatively connected (blue dashed edges).

### Subtypes in diseased AP patients

At the genus level, the AP patients can be partitioned into two groups (labelled as A and B, respectively), using an R package *hclust* (see Additional file 1: Figure S3). This clustering showed significant agreement with the clinical classification based on the lesion area of AP, where A and B match to LSA and SSA with an accuracy of 0.885 (*p* < 10^(− 6)), respectively (See Table 1, complete clinical information was in Additional file 4). At phylum level, a big difference between A and B was found in the percentages of *Protepbacteria* (19.6% in A and 32.5% in B) and *Actinobacteria* (14.9% in A and 6.1% in B), see Additional file 1: Figure S5. Differential analysis of A versus B identified 53 significant OTUs, basically from phyla *Proteobacteria*, *Firmicutes*, *Actinobacteria and Bacteroidetes,* details see Additional file 5. Interestingly, 51 of them showed higher abundance in group B, see Additional file 1: Figure S6, and showed different variation patterns, **P1~P4**. That means these bacteria enriched in the teeth with small lesion area (SSA). In other word, the lesion area will be bigger without these bacteria. Furthermore, a large number of B-abundant OTUs showed similar abundance in the healthy teeth, yellow rectangle in Additional file 1: Figure S6. Among them, *lactococcus* (genus), known as a lactic acid bacteria, was reported to cause teeth decay known as caries. However, previous researches demonstrated that fermentation products produced by lactic acid bacteria contribute to the attenuation of pathogens and improvement of human animal health [39]. We therefore speculate that the abundant lactic acid bacteria in our case (*Lactococcus*, *Enterococcus* and *Streptococcus*) might act as probiotics to impair the infection.

**Table 1:** Clinical information of the AP patients without RCT treatment

SampleID	Gender	Age	Lesion	Levels	Clusters
T_D_None_B.24	M	74	2×2 mm	SSA	B
T_D_None_B.23	F	62	2×2 mm	SSA	B
T_D_None_A.3	M	72	2×3 mm	SSA	A
T_D_None_A.25	M	65	3×2 mm	SSA	A
T_D_None_B.2	F	41	3×2 mm	SSA	B
T_D_None_B.15	F	52	3×2 mm	SSA	B
T_D_None_B.1	F	74	3×3 mm	SSA	B
T_D_None_B.27	F	72	3×3 mm	SSA	B
T_D_None_B.30	F	62	3×3 mm	SSA	B
T_D_None_A.14	M	45	4x3mm	SSA	A
T_D_None_A.26	M	28	3×4 mm	SSA	A
T_D_None_B.7	F	62	4×3 mm	SSA	B
T_D_None_B.22	M	79	4×4 mm	SSA	B
T_D_None_B.21	F	34	4×4 mm	SSA	B
T_D_None_A.28	M	59	6×3 mm	LSA	A
T_D_None_A.9	F	73	4×5 mm	LSA	A
T_D_None_A.12	F	29	6×4 mm	LSA	A
T_D_None_A.8	F	52	6×5 mm	LSA	A
T_D_None_A.19	F	61	5×6 mm	LSA	A
T_D_None_A.29	M	34	6×5 mm	LSA	A
T_D_None_A.5	M	41	8×5 mm	LSA	A
T_D_None_A.Xy	F	42	8×5 mm	LSA	A
T_D_None_A.13	M	83	6×7 mm	LSA	A

### Effect of RCT on bacterial communities

No significant OTUs was identified between NRCT and RCT. Decreased OTUs due to AP showed no obvious changes after RCT (Fig. 5a). In some patients (eg. T_D_RCT.Jm), most of the increased OTUs (due to AP) did disappeared, failure of the therapy might be due to the fact that decreased OTUs are not recovered. From this, we say that AP might be a multi-species infection.

## Discussion

In this study, we collected microbiota samples from root apex diagnosed as AP with and without RCT treatment, as well as the healthy controls including root apex and supragingival buccal biofilm, and sequenced the 16S ribosomal RNA gene fragments using a next-generation sequencing platform.

Our study focused on the bacterial community around the root apex to investigate the microbial features with respect to apical periodontitis. We start our investigations from the community richness and diversity in AP patients (NRCT) as well as other relevant groups, including failed AP after root canal treatment (RCT), healthy control of root apex (HT), as well as and the oral healthy control (HO). We observed significant decreasing of the richness and diversity from healthy controls to diseased samples (Fig. 1), somehow indicates that AP has broken the microbial balance, where both richness and diversity were reduced. Next, we identified the key bacteria relevant to AP by comparison between AP without root canal treatment (NRCT) and the healthy control (HT). Compared to the healthy root apex, we found a large population of bacteria down-regulated in AP root apex, basically from genera of *Haemophilus* (*parainfuenzae* in particular), *Streptococcus*, *Granulicatella*, *Actinomyces*, and some unknown bacteria, as well as the species including *subflava*, *dispar*, *nanceiensis*, *melaninogenica*.

Our study emphasizes the importance of the healthy control, based on which we revealed plentiful of bacteria around healthy root apex and further identified both up and down regulated bacteria relevant for AP. Furthermore, we involved the supragingival buccal biofilm (HO) as additional healthy control to confirm whether it works instead of the healthy root apex (HT). Global view of the clustering of all the 16S samples using R package *hclust* showed that HO samples are much closer to HT samples than to AP teeth samples (NRCT or RCT), see Additional file 1: Figure S4. Meanwhile, microbial richness and diversity are also similar with higher values in HO and HT samples, and no significant OTUs can be found through differential analysis between HT and HO. However, these are not enough to determine that supragingival buccal biofilm can work as healthy controls. To confirm this we did differential analysis between NRCT and HO, and found lots of bacteria (150 OTUs) significantly up/down regulated. Nevertheless, only 13 out of 150 OTUs overlapped with that of NRCT versus HT. Most of the decreased OTUs and some wildly reported increased OTUs including *Sphaerochaeta*, *Burkholderia*, *Schwartzia*, *Dialister* and [*Prevotella*], missed in NRCT versus HO*.*

## Conclusions

This study provide a global view of the microbial community of the apical periodontitis associated cohorts, and revealed that apical periodontitis involved not only bacteria accumulated with a high abundance, but also those significantly reduced ones due to microbial infection. This provides a more precise spectrum of pathogenic community, aiding for choosing or designing antimicrobial agent used in RCT treatment.

## Additional files

Additional file 1: Packages of the additional figures. (PDF 609 kb)

Additional file 2: Differential analysis results between AP samples (NRCT) and healthy teeth (HT), at OTU level. (XLSX 29 kb)

Additional file 3: Differential analysis results between AP samples (NRCT) and healthy teeth (HT), at Genus level. (XLSX 12 kb)

Additional file 4: Clinical information of all the 50 samples. (XLSX 15 kb)

Additional file 5: Differential analysis results between subtypes A and B inNRCT at OTU level. (XLSX 22 kb)

## Abbreviations

AP: Apical periodontitis
OTU: Operational taxonomic unit
RCT: Root canal treatment
tSNE: t-distributed stochastic neighbor embedding
