# Supplementary material for: Microbiota in the apical root canal system of tooth with apical periodontitis
Source: BMC Genomics. 2019 Apr 4;20(Suppl 2):189. doi: 10.1186/s12864-019-5474-y (PMC6456935; doi:10.1186/s12864-019-5474-y)
Supplement: Supplementary file 1 — Packages of the additional figures. (PDF 609 kb) [file 12864_2019_5474_MOESM1_ESM.pdf]

All additional figures are packaged in this file.

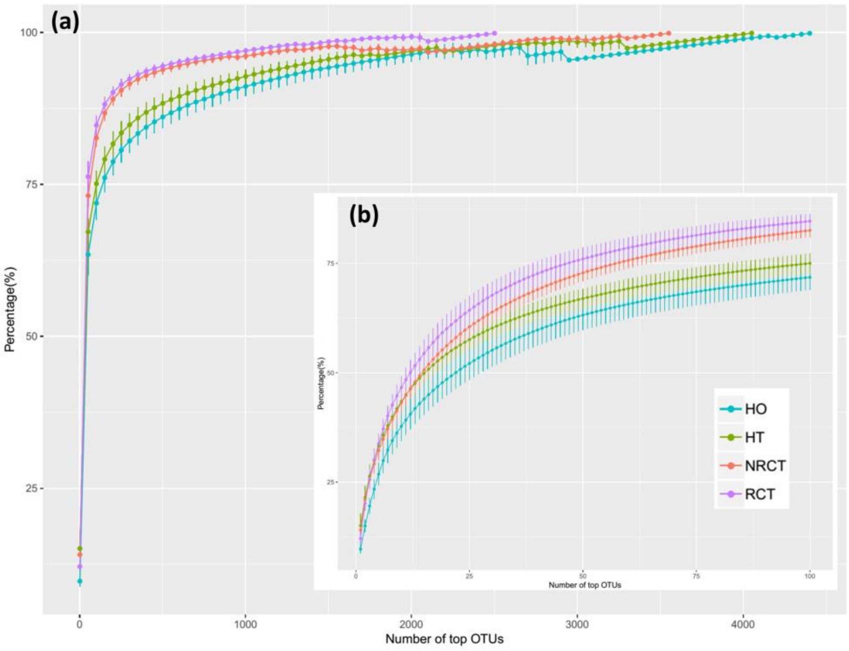

Figure 1 Dynamics of the fraction of the abundance of top OTUs in whole individual sample in different groups.(a) The dynamics of the fraction of top OTUs with all OTUs considered; (b) a zoom out of the initial stage where top 100 OTUs involved. NRCT, AP without RCT treatment; RCT, AP failed after RCT treatment; HT, Healthy control of root apex; HO, the oral healthy control.

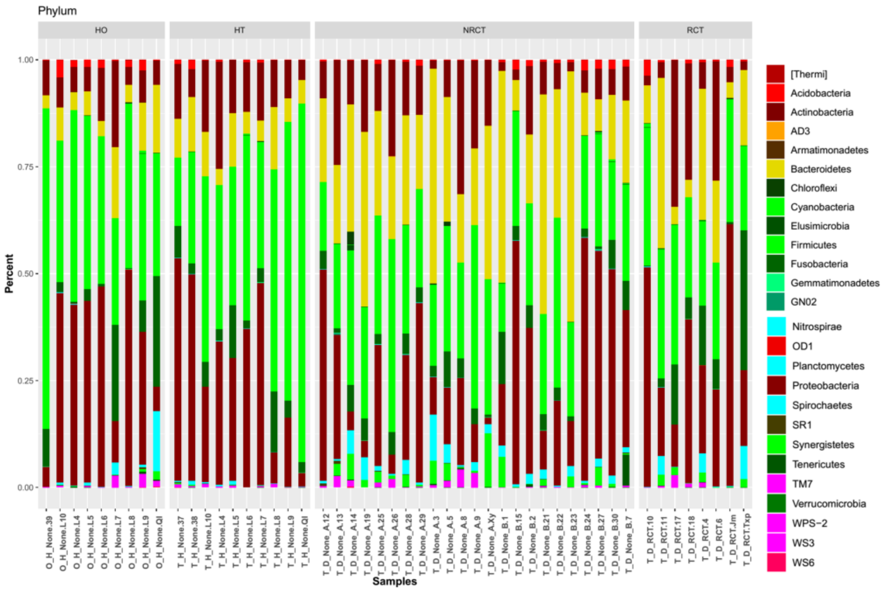

Figure 2 Distribution of percentage of the species in individual samples at phylum level. NRCT, AP without RCT treatment; RCT, AP failed after RCT treatment; HT, Healthy control of root apex; HO, the oral healthy control.

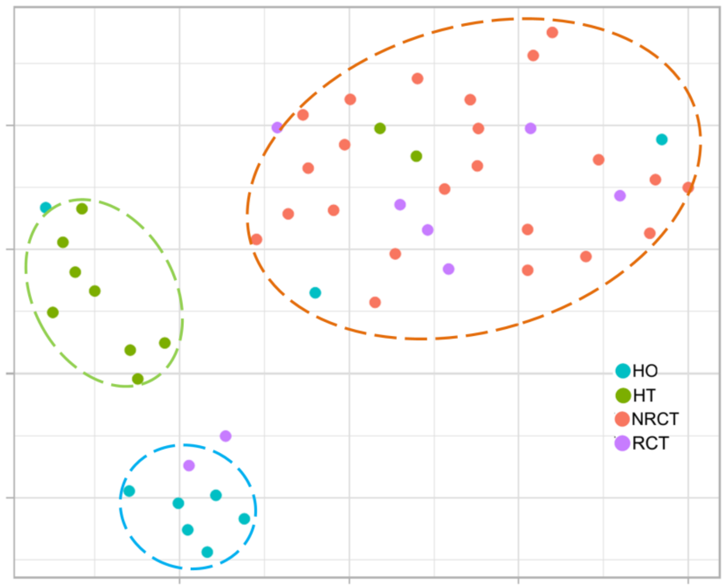

Figure 3 tSNE plot of the 50 samples. NRCT, samples in AP without RCT treatment; RCT, samples in AP failed after RCT treatment; HT, samples in healthy control of root apex; HO, samples in the oral healthy control.

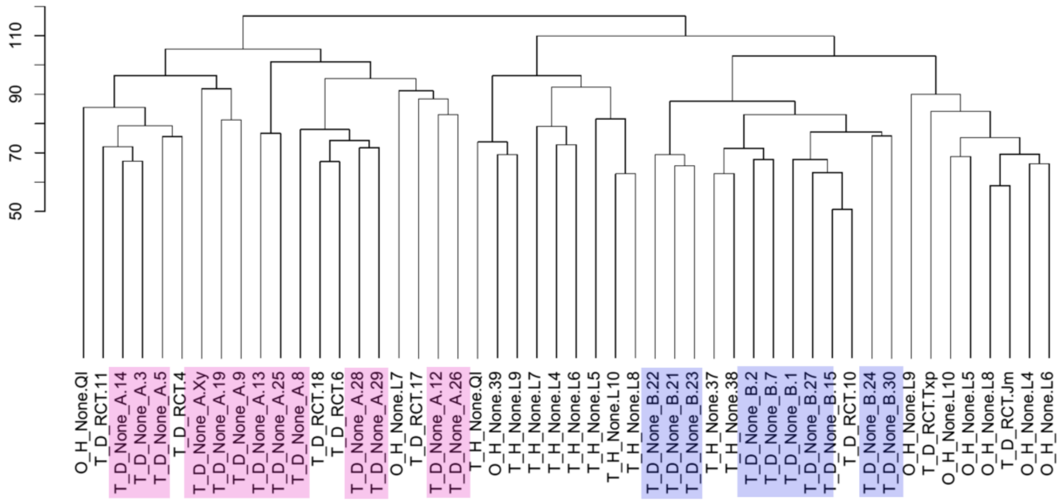

Figure 4 Clustering of the all 50 sample using R package *hclust.* NRCT samples are separately distributed into two parts, highlighted with pink and light blue for A and B, respectively.

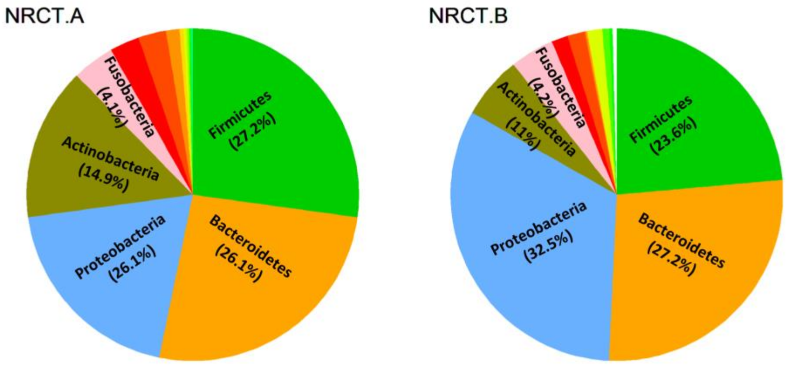

Figure 5 Comparisons of the distributions at the phyla level between subtypes A and B in NRCT samples.

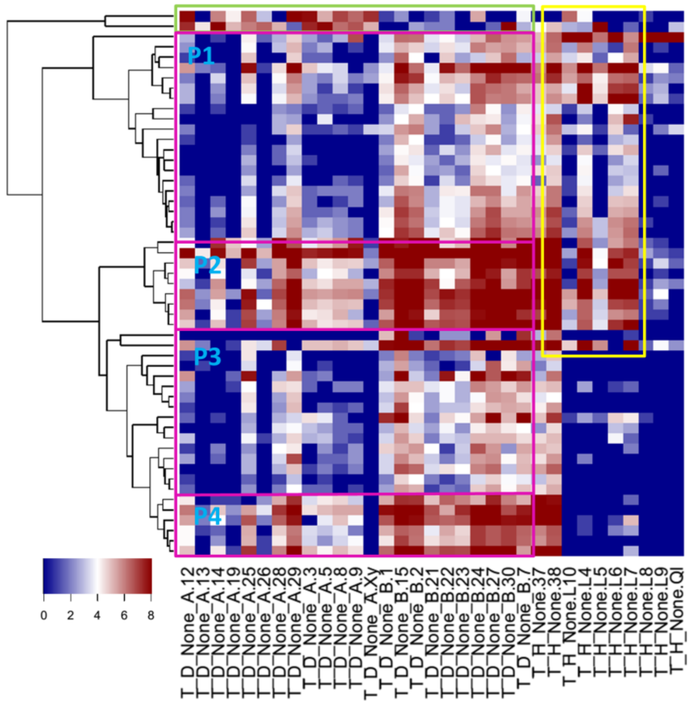

Figure 6 Heatmap of the key OTUs resulted from comparison between subtypes A and B. Based on the abundance of the samples from A and B as well as healthy teeth, these genes are clustered into several parts. Higher in A: green rectangle; Higher in B: pink rectangle. P1 and P2 are OTUs higher in B, and have a high abundance in healthy samples, but differed at abundance level; P3 and P3 are also OTUs higher in B, differed at abundance level, respectively, and showed empty in healthy samples. Yellow rectangle is to highlight the healthy samples having abundance.
